# Profiling and Preparation of Metabolites from Pyragrel in Human Urine by Online Solid-Phase Extraction Coupled with High Performance Liquid Chromatography Tandem Mass Spectrometry Followed by a Macroporous Resin-Based Purification Approach

**DOI:** 10.3390/molecules22030494

**Published:** 2017-03-21

**Authors:** Xin Zhao, Jingjing Jiang, Guang Yang, Jie Huang, Guoping Yang, Guangwei He, Zhaoxing Chu, Taijun Hang, Guorong Fan

**Affiliations:** 1 Department of Pharmaceutical Analysis, School of Pharmacy, China Pharmaceutical University, Nanjing 210009, China; yoyo0132@163.com; 2 Shanghai Key Laboratory for Pharmaceutical Metabolite Research, School of Pharmacy, Second Military Medical University, Shanghai 200433, China; jiangjingj8@163.com (J.J.); diabloyg@126.com (G.Y.); 3 Center of Clinical Pharmacology, Third Xiangya Hospital, Central South University, Changsha 410013, Hunan, China; cellahuang1988@163.com (J.H.); ygp9880@126.com (G.Y.); 4 Hefei Institute of Pharmaceutical Industry Co., Ltd., Wenshan Road, Hefei 230601, China; hgwhipi@hotmail.com (G.H.); chuzhaoxing@163.com (Z.C.); 5 Department of Clinical Pharmacy, Shanghai General Hospital, School of Medicine, Shanghai Jiao Tong University, No. 100 Haining Road, Shanghai 200080, China; 6 Laboratory of Drug Metabolism & Pharmacokinetics, School of Medicine, Tongji University, No. 1239 Siping Road, Shanghai 200092, China

**Keywords:** Pyragrel, metabolite, human urine, online SPE-HPLC-MS^n^, macroporous resin, PHPLC

## Abstract

Pyragrel, a new anticoagulant drug, is derived from the molecular combination of ligustrazine and ferulic acid. Pyragrel showed significant inhibitory activity against platelet aggregation induced by adenosine diphosphate (ADP), and had been approved for a phase I clinical trial by CFDA. To characterize the metabolites of Pyragrel in human urine after intravenous administration, a reliable online solid-phase extraction couple with high performance liquid chromatography tandem mass spectrometry (online SPE-HPLC-MS^n^) method was conceived and applied. Five metabolites were detected and tentatively identified, which suggested that the major metabolic pathways of Pyragrel in human were double-bond reduction, double-bond oxidation, and then followed by glucuronide conjugation. Two main metabolites were then prepared using β-glucuronide hydrolysis and macroporous resin purification approach followed by preparative high-performance liquid chromatography (PHPLC) method, with their structures confirmed on the basis of nuclear magnetic resonance (NMR) data. This study provided information for the further study of the metabolism and excretion of Pyragrel.

## 1. Introduction

Cardio-cerebrovascular disease is frequent and common in the aging population, as well as imperiling younger patients in recent years. Thrombus formation at the site of atherosclerotic lesions, especially on a ruptured plaque, plays an essential role in the atherothrombosis, inducing ischemic complications which may cause a variety of serious clinical syndromes such as stroke, acute myocardial infarction (AMI), acute coronary syndrome (ACS) and peripheral arterial disease and lead to mortality or disability. The use of antiplatelet therapy aimed for reducing vascular events has been extensively studied for many years. For instance, in early years the ‘gold standard’ for ACS treatment involved the combination aspirin and clopidogrel by oral administration [1]. In recent years, new antiplatelet agents in development such as prasugrel, ticagrelor and cangrelor, which showed improved pharmacokinetic and pharmacodynamic features, are anticipated to alleviate medication risks and improve clinical outcomes [2,3,4].

**Figure 1:**
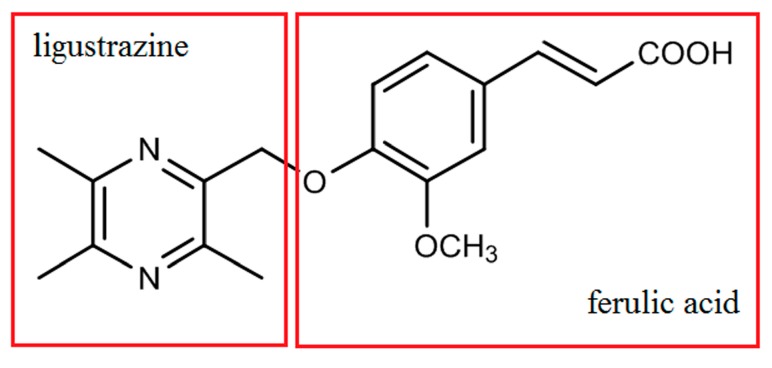
Chemical structure of Pyragrel.

Rhizoma chuanxiong is a famous traditional Chinese medicine whose pharmacological activity is described as positive hemodynamic effect [5]. Ligustrazine (tetramethylpyrazine, TMP) and ferulic acid as well as several other components were identified as the active compounds responsible for the holistic treatment effect of R. chuanxiong [6,7,8,9,10,11]. Pyragrel (Figure 1) is a synthetic compound derived from the molecular combination of ligustrazine and ferulic acid. Pharmacodynamic studies of Pyragrel demonstrated that it could exert activities such as expansion of blood vessels, reduction of blood viscidity and alleviation of cerebral ischemia [12]. Pharmacological studies have revealed that Pyragrel showed significant inhibitory activity against platelet aggregation induced by adenosine diphosphate (ADP) [13,14,15], and the mechanism was inferred as the inhabitation of thromboxane A2 and relieving inflammatory injury of endothelial cells through NF-κB signal pathway [16]. At present, a phase I clinical trial has already been performed successfully on twelve healthy volunteers, and it has been revealed that Pyragrel is mainly excreted through the urinary pathway. However, currently no study related to the metabolism of Pyragrel has been performed, and no information of metabolites of Pyragrel has been acquired, which may have influence on the total efficacy and toxicity in the treatment. Thus it is essential to establish a reliable method for the identification and quantification of Pyragrel and its major metabolites in human urine.

During the identification of drug metabolites, sample pretreatment is a critical step including purification and enrichment. Solid-phase extraction (SPE) as common approach was a powerful tool, but may suffer from such limitations as solvent and time consumption as well as tedious manual operation. Online solid-phase extraction as an improved SPE approach significantly facilitated the sample pretreatment step and avoided introduction of contamination in manual operation, thus suitable for the pretreatment of biological samples and has been applied in the metabolism studies [17,18].

To further confirm the structures of the main metabolites and acquire the excretion information, it is essential to obtain adequate reference substances. Since human urine is a complex matrix containing endogenous components, it is not appropriate to directly introduce urine samples to the preparation procedure before some extra pretreatment is done. The macroporous resin purification method as an effective approach, that accepts loading of samples of relatively large volume, and is suitable for rough purification of urine sample [19,20].

In this study, an online solid-phase extraction coupled with high-performance liquid chromatography tandem mass spectrometry approach was constructed and applied in the identification and profiling of metabolites of Pyragrel in human urine. Five metabolites were tentatively identified through the fragmentation patterns displayed in tandem mass spectrometry, and their metabolic pathways are discussed. Two main metabolites were prepared using β-glucuronide hydrolysis and macroporous resin purification approach followed by preparative high-performance liquid chromatography (PHPLC) method, with their molecular structures confirmed on the basis of nuclear magnetic resonance (NMR) data. This study provided information for the further study of the metabolism and excretion of Pyragrel.

## 2. Results and Discussion

### 2.1. Identification of Metabolites in Human Urine

#### 2.1.1. Optimization of Online SPE-HPLC and MS Conditions

Sample pretreatment and separation were accomplished using the online SPE-HPLC system. The optimization of SPE was conducted aimed to remove endogenous interferences from the urine matrix, retain the target analytes and obtain a stable recovery. In this study the molecular polarity of the metabolites in urine varied from polar to low-polar and therefore polar/non-polar balanced polymeric sorbents were considered in SPE procedure. Meanwhile, rapid analysis with acceptable separation efficiency was preferred in the HPLC analysis procedure. In this work, Hypersil GOLD PFP (20 × 2.1 mm i.d., 12 µm, Thermo Fisher Scientific, Waltham, MA, USA) gave the most favorable performance in cleanup and enrichment for the polarity-diversed analytes, and a Boltimate™ core-shell C18 column (100 mm × 4.6 mm i.d., 2.7 µm, Welch Materials Inc., Shanghai, China) was selected as the analysis column. Acetic acid and ammonium acetate was added to the mobile phase to enhance the retention of Pyragrel and its metabolites as well as to improve the separation and peak shape. The SPE procedure was performed in gradient elution mode to remove those endogenous components of high polarity, and those endogenous components of low polarity was washed out after the introduction of analytes to the analytical column. As for MS conditions, the full scan was performed in both positive and negative ionization modes, and the result demonstrated that positive ionization mode showed higher sensitivity for the metabolites from Pyragrel. Several parameters including ion spray voltage, capillary voltage and tube lens offset in IT MS as well as capillary voltage, fragmentor voltage and skimmer voltage in TOF MS were then optimized with the injection of neat solution of Pyragrel.

#### 2.1.2. Identification of Pyragrel and Its Metabolites

**Figure 2:**
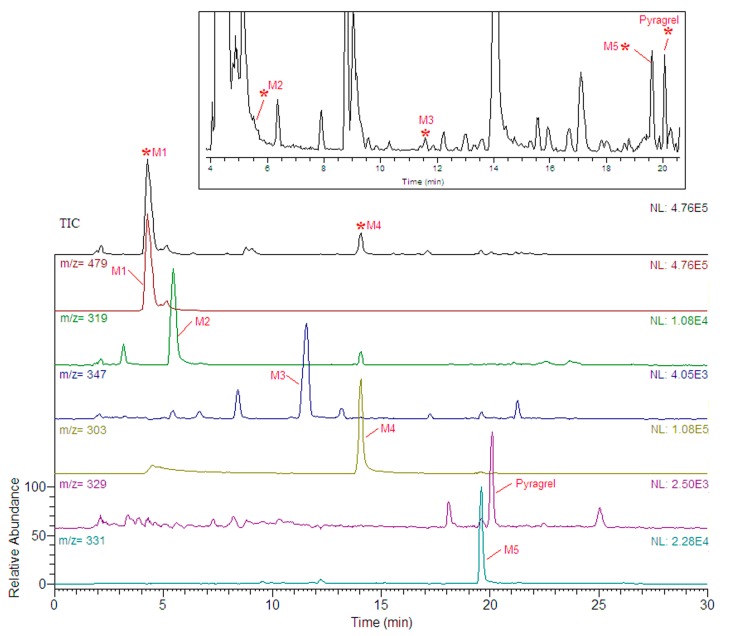
Total and extracted ion current chromatograms for metabolites of Pyragrel in human urine by HPLC-MS^n^ analysis.

**Figure 3:**
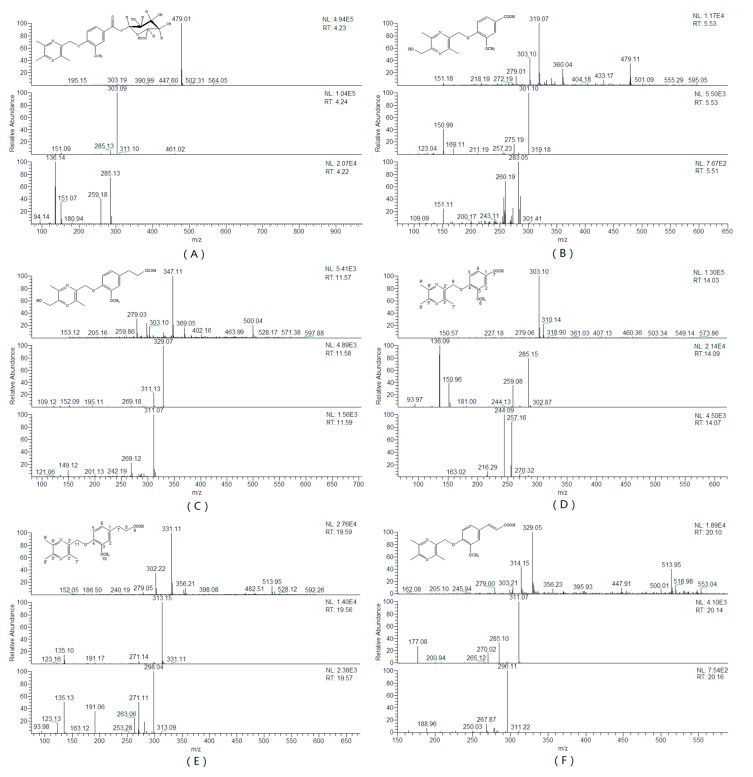
The mass spectra of metabolites in positive ion mode of M1 (**A**), M2 (**B**), M3 (**C**), M4 (**D**), M5 (**E**) and parent drug Pyragrel (**F**).

Human urine samples were analyzed under optimized condition. Five metabolites were detected by comparison of the extracted ion information with blank urine sample. The possible metabolites were analyzed by MS^n^ spectra for the further confirmation of their structures. MS information of the metabolites is given in Table 1, and their extracted ion chromatograms and tandem mass spectra are shown in Figure 2 and Figure 3, respectively.

**Figure 4:**
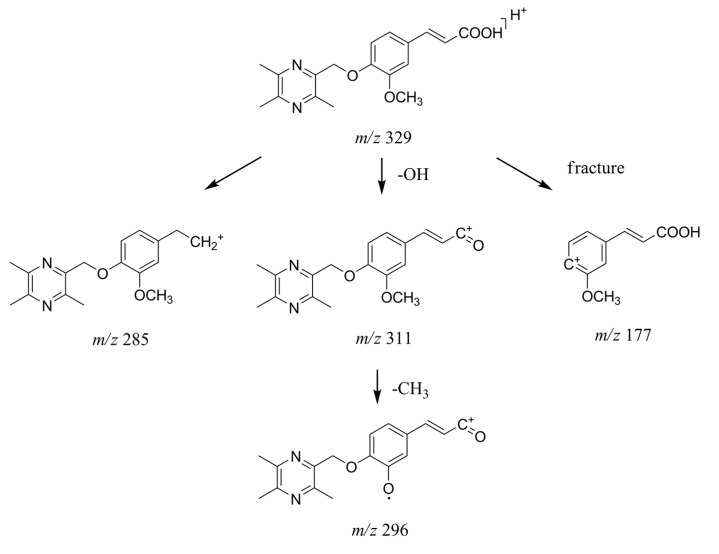
Proposed fragmentation patterns of Pyragrel.

The parent drug Pyragrel was observed in urine at a retention time of 20.8 min showing [M + H]^+^ at *m*/*z* 329. The fragment ions *m*/*z* 311, 18 Da less than the quasi-molecular ions, correspond to the product after dehydroxylation on the carboxyl group. Another fragment ions *m*/*z* 285, 44 Da less than the major parent ions, were inferred as the product after loss of the carboxyl group. A minor fragment ions *m*/*z* 177, were supposed to be the product after fracture of the C–O bond between the oxygen atom and the benzene ring. The MS^3^ fragment ions *m*/*z* 296 from major product ions *m*/*z* 311 was inferred to be formed by loss of methyl group [21]. The fragmentation patterns of Pyragrel inferred in Figure 4 could offer a basis to deduce the molecular structures of the metabolites.

It was observed that the quasi-molecular ions of M4 (*m*/*z* 303) was detected at the retention time of 14.03 min, 26 Da less than those of Pyragrel (*m*/*z* 329), and corresponds to the product after loss of two carbon atoms and two hydrogen atoms. In consideration of the fact that the unsaturated double bond can be oxidized, it was inferred that the formation of M4 involved oxidation of double bond, formation of vicinal diol, fracture of C–C bond and formation of aromatic carboxylic acid.

The major fragment ions *m*/*z* 285, 18 Da less than the quasi-molecular ions, were inferred as the product after dehydroxylation of the carboxyl group, which was similar from the fragmentation pattern of Pyragrel. Another major fragment ion at *m*/*z* 259, 44 Da less than the quasi-molecular ions, was inferred as the product after loss of the carboxyl group on benzene ring. The fragment ion *m*/*z* 151 were supposed to be the product after fracture of the C–O bond similar as the situation of Pyragrel. The MS^3^ fragment ions *m*/*z* 257 from major product ions *m*/*z* 285, by loss of 28 Da, corresponded to the product after loss of a carbonyl group. The molecular structure of M4 was then inferred as shown in Figure 3D, and the metabolic reaction between Pyragrel and M4 was thought to be double bond oxidation followed by formation and oxidation of the vicinal diol on the structure of ferulic acid.

The quasi-molecular ion of M5 (*m*/*z* 331) was detected at the retention time of 19.59 min, 2 Da more than those of Pyragrel (*m*/*z* 329), and corresponds to the product after double-bond reduction. The major fragment ions *m*/*z* 313, 18 Da less than the quasi-molecular ions, were inferred as the product after dehydroxylation of the carboxyl group, which was similar from the fragmentation patterns of Pyragrel and M4. The MS^3^ fragment ions *m*/*z* 298, 15 Da less than the MS^2^ product ions, were inferred to be formed by loss of methyl group similar as situation of Pyragrel. The MS^3^ fragment ions *m*/*z* 191, 122 Da less than the MS^2^ product ions, were supposed to be the product after loss of the trimethylpyrazine group. Another MS^3^ fragment ion *m*/*z* 135 were inferred as the tetramethylpyrazine ion. The molecular structure of M5 was then inferred as showed in Figure 3E, and the biological metabolism from Pyragrel to M5 was inferred as double-bond reduction on the structure of ferulic acid.

The quasi-molecular ion of M1 (*m*/*z* 478), detected at the retention time of 4.23 min, was 176 Da more than M4 (*m*/*z* 303) and one of the produced fragment ions was [M + H − C_6_H_8_O_6_]^+^ (*m*/*z* 303). Meanwhile M1 showed weaker retention trend compared with other metabolites. After hydrolysis with β-glucuronidase, there was an increase to the peak area of metabolite M4, and a decrease to the peak area of M1. Therefore, it was inferred that M1 was the glucuronide conjugate of M4 (Figure 3A). The MS^3^ fragmentation patterns of the product ions *m*/*z* 303 confirmed the inference since it was almost the same as those of the quasi-molecular ions of M4 (*m*/*z* 303).

The quasi-molecular ion of metabolite M2 (*m*/*z* 319), detected at 5.53 min, was 16 Da more than M4 (*m*/*z* 303), indicating that oxidation may happened during the formation of M2 from M4. The major fragment ions *m*/*z* 301, 18 Da less than the quasi-molecular ions, were inferred as the product after dehydroxylation of the carboxyl group similar as the situation of M4. The fragment ions *m*/*z* 275 and *m*/*z* 151 also corresponded to the fragmentation pathway of M4, as inferred through the loss of carboxyl group and the fracture of C–O bond, respectively. Besides, since M2 and M4 shared the same product ions as *m*/*z* 151, indicating that the oxidation from M4 to M2 was not happened on the aromatic acid group, it was then inferred that the oxidation was happened on the tetramethylpyrazine group, probably on the methyl group. The molecular structure of M2 was then inferred as showed in Figure 3B, and the fragmentation patterns was inferred as those in supplementary materials Figure S1.

Similarly, the quasi-molecular ion of metabolite M3 (*m*/*z* 347), detected at the retention time of 11.57 min, was 16 Da more than those of M5 (*m*/*z* 331). The MS^2^ product ions *m*/*z* 329 also corresponded to the fragmentation patterns of M5 as well as other metabolites. The major MS^3^ product ions *m*/*z* 311, 18 Da less than the ions *m*/*z* 329, were thought to be the product after loss of carbonyl group followed by cyclization. Another MS^3^ product ions *m*/*z* 269 were then inferred as the product of *m*/*z* 329 after loss of carbonyl group and methoxy group. The molecular structure of M3 was then inferred as showed in Figure 3A, and the fragmentation patterns was inferred as those in Figure S2.

**Figure 5:**
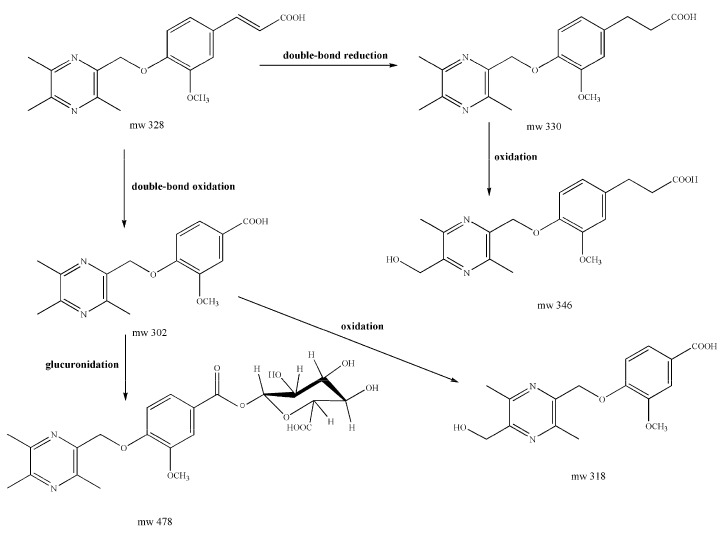
Proposed major metabolic pathway of Pyragrel in human urine.

Figure 5 shows the proposed major metabolic pathway of Pyragrel in human urine. M1, M4 and M5 were the main metabolites, suggesting that double-bond reduction, double-bond oxidation followed by glucuronide conjugation were the major metabolic pathways for Pyragrel. In order to further prove the correctness of metabolite structures, M4 and M5 were prepared from human urine through macroporous resin approach followed by preparative high-performance liquid chromatography.

### 2.2. Preparation of Major Metabolites

Five metabolites from Pyragrel were tentatively identified in human urine, among which M4 and M5 were recognized as major ones. To further confirm the molecular structure of M4 and M5, a preparation step involving glucuronide hydrolysis and macroporous resin purification approach followed by preparation using PHPLC was applied.

#### 2.2.1. Hydrolysis Reaction

Since the metabolite M1 were identified as the glucuronide conjugate of M4, the collected urine sample was first subjected to hydrolysis before purification step. 100 µL β-glucurnoidase (10^5^ unit mL^−1^) was added to 6 mL urine sample under 37 °C for hydrolysis reaction, and 200 µL urine samples were collected at the time point 2 h, 4 h, 6 h, 8 h, 12 h and 24 h, and subjected to HPLC analysis for the evaluation of effect of hydrolysis reaction time. It was showed that during the first two hours significant increase was observed to the peak area of M4, while after four hours of reaction no tread was observed in the peak area of M4. It was then inferred that the hydrolysis reaction was almost completed in four hours. The hydrolysis reaction system was then scaled for practice. 5 mL β-glucurnoidase was added to 300 mL urine sample for hydrolysis reaction, and it was observed that the reaction was almost done in four hours since after that no increase to the peak area of M4 was observed. The hydrolysis reaction time was then selected as four hours.

#### 2.2.2. Macroporous Resin Purification

Since human urine is complex samples containing endogenous components, it is not appropriate to directly introduce sample to preparation step before an extra pretreatment is done. A clean-up step based on macroporous resin purification approach was applied for the removal of β-glucurnoidase and endogenous components in urine. The D-101 macroporous resin was tested and selected for the rough purification of urine sample. After loading of urine samples, the column was allowed for absorption process lasting one hour. The column was firstly washed using deionized water for the removal of β-glucurnoidase and endogenous components. After that, the column was slightly washed using 40% (*v*/*v*) ethanol as buffer between aqueous and organic eluent solvent to prevent damage to the resin. The column was then subjected to desorption elution using 95% (*v*/*v*) ethanol. The collected eluent was pooled, concentrated to dryness and resolved in 50% (*v*/*v*) acetonitrile for further preparation in PHPLC.

#### 2.2.3. Preparative High-Performance Liquid Chromatography

For analytical purposes, the chromatographic optimization usually aims for better separation, where gradient elution mode was commonly applied. However, for preparative purposes it is necessary to be aware of the analysis time per injection. Since the re-equilibrium process cannot be avoided in gradient elution mode, isocratic elution mode was preferred in this study. Besides, formic acid instead of acetic acid/ammonium acetate was added to the mobile phase to facilitate the posttreatment procedure.

**Figure 6:**
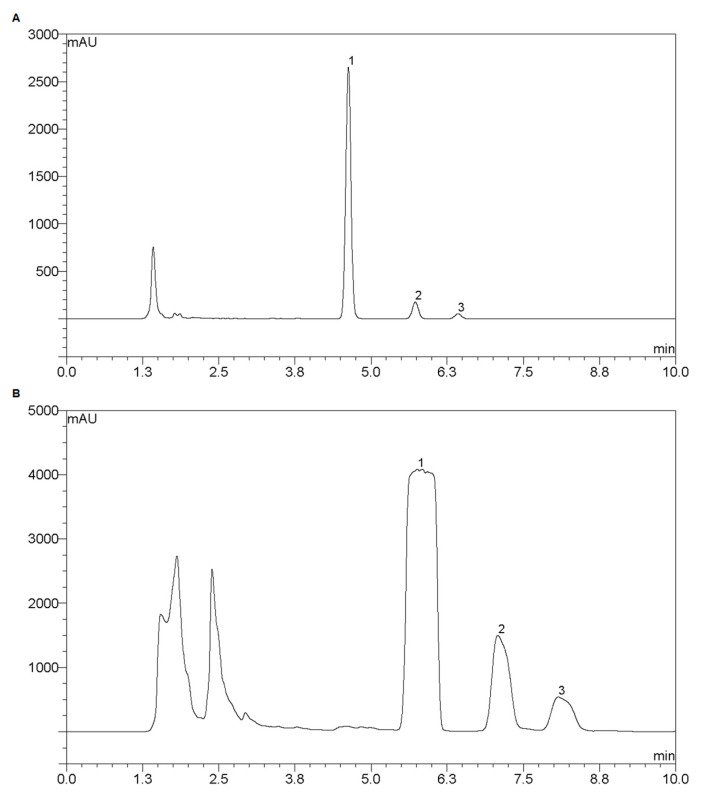
Chromatographic plots of metabolites from Pyragrel in analytical condition (**A**) and preparative condition (**B**). Peak No., 1—M4, 2—M5, 3—Pyragrel.

The optimization was then carried out through a scale-up approach using a pair of columns equipped with the same stationary phase, where the chromatographic performance of the analytical column was utilized to predict the chromatographic performance by the preparative column under scaled condition. The analytical chromatographic separation was conducted using an YMC-Pack Pro C18 RS column (150 mm × 4.6 mm i.d., 5 µm, 8 nm, YMC Co., Ltd., Kyoto, Japan) at 45 °C. The mobile phase in isocratic mode was optimized as 35% acetonitrile and 65% water (containing 0.1% formic acid) with a flow rate of 1 mL·min^−1^, and the injection volume was 10 µL. The chromatographic plot was shown in Figure 6A.

The preparative chromatographic separation was then conducted using an YMC-Pack Pro C18 RS column (150 mm × 10.0 mm i.d., 5 µm, 8 nm, YMC Co., Ltd.) under scaled condition. The flow rate was 4 mL·min^−1^, and the injection volume was 1 mL. The chromatographic plot was shown in Figure 6B with acceptable separation effect.

Under the optimized condition, approximately 40 mg of M4 and 5 mg of M5 were prepared using 300 mL urine samples. The ^1^H- and ^13^C-NMR data of M4 and M5 were as follows, and the NMR spectra are shown in Figures S3–S6.

M4, ^1^H-NMR (600 MHz, MeOD): δ 7.64 (1H, dd, *J* = 8.4, 2.0 Hz, H-5), 7.57 (1H, d, *J* = 2.0 Hz, H-2), 7.16 (1H, dd, *J* = 8.3, 4.0 Hz, H-6), 5.24 (2H, s, H-9), 3.84 (3H, s, H-8), 2.57 (3H, s, H-7′), 2.52 (3H, s, H-8′), 2.51 (3H, s, H-9′); ^13^C-NMR (151 MHz, MeOD): δ 168.20 (C-7), 152.03 (C-4), 151.50 (C-3), 150.07 (C-2′), 149.26, 148.98, 145.62 (C-3′/C-5′/C-6′), 123.73 (C-6), 123.33 (C-1), 112.78 (C-2), 112.49 (C-5), 70.07 (C-9), 54.98 (C-8), 19.93, 19.72, 18.91 (C-7′/C-8′/C-9′) (Figure 3D).

M5, ^1^H-NMR (600 MHz, MeOD): δ 6.97–6.94 (m, 1H, H-6), 6.86 (1H, d, *J* = 1.9 Hz, H-2), 6.73 (1H, dd, *J* = 8.1, 1.8 Hz, H-5), 5.12 (2H, s, H-11), 3.79 (3H, s, H-11), 2.85 (2H, t, *J* = 7.6 Hz, H-8), 2.59–2.56, (5H, m, H-7/H-7′), 2.51 (3H, s, H-8′), 2.50 (3H, s, H-9′); ^13^C-NMR (151 MHz, MeOD): δ 175.35 (C-9), 151.20 (C-4), 150.14 (C-3), 150.01(C-2′), 148.79, 146.32, 146.23 (C-3′/C-5′/C-6′), 135.21 (C-1), 120.04 (C-6), 115.29 (C-2), 112.34 (C-5), 70.71 (C-11), 54.89 (C-10), 35.49 (C-8), 30.26 (C-7), 19.89, 19.64, 18.93 (C-7′/C-8′/C-9′) (Figure 3E).

## 3. Experimental Section

### 3.1. Chemicals and Reagents

The reference substance of Pyragrel sodium with purity above 99% were synthesized by Hefei Institute of Pharmaceutical Industry Co., Ltd. (Hefei, China). The β-glucuronidase (10^5^ unit mL^−1^) was purchased from Sigma-Aldrich (St. Louis, MO, USA). HPLC grade formic acid, acetic acid, acetonitrile and ammonium acetate were obtained from Tedia (Fairfield, CA, USA). Deionized water (18.2 MΩ/cm) was generated through a Milli-Q system from Millipore (Bedford, MA, USA).

D-101 macroporous resin was purchased from Shanghai Yuanye Bio-Technology Co., Ltd. (Shanghai, China). The resin was washed, soaked for 12 h using 95% (*v*/*v*) ethanol and washed extensively using water before experiments for the removal of monomers and residual porogenic agents.

### 3.2. Urine Samples

Urine samples were obtained from healthy volunteers belonged to a clinical trial conducted by the Third Xiangya Hospital of Central South University (Changsha, China). All twelve healthy volunteers signed informed-consent forms approved by the ethics committee of the hospital. Urine samples were collected before and 0–3 h/3–7 h after oral administration of 240 mg Pyragrel. The urine samples were then centrifuged, and the supernatant was divided into centrifuge tubes and stored at −80 °C until analysis.

### 3.3. SPE-HPLC-MS^n^ Instrumentation

SPE-HPLC analysis was performed on a Dionex Ultimate 3000 Liquid Chromatography system (Thermo Fisher Scientific) equipped with degasser, dual-gradient pump, an auto-sampler with a 100 µL sample loop, column oven with a six-port valve and a diode array detector (DAD). Online cleanup/enrichment was performed using a Hypersil GOLD PFP (20 mm × 2.1 mm i.d., 12 µm, Thermo Fisher Scientific) as trap column. 100 µL of the prepared urine sample was loaded onto the trap column. After online cleanup and enrichment, separation of the analytes was achieved using a Boltimate™ core-shell C18 column (100 mm × 4.6 mm i.d., 2.7 µm, Welch Materials Inc., Shanghai, China) at 40 °C, and the detection wavelength was 281 nm. The mobile phase consisted of Acetonitrile and water (containing 0.4% acetic acid and 25 mM ammonium acetate), and the elution procedures was listed in Table 2.

The LCQ Fleet Mass Spectrometer (Thermo Fisher Scientific) equipped with an ESI ion source was used for identification of Pyragrel and its metabolites. Data acquisition and processing was performed using Xcalibur™ 2.6.0 workstation (Thermo Fisher Scientific). Full scan was performed in the positive mode with a mass range from *m*/*z* 150 to 600 amu with data-dependent scan mode, where ions with higher intensity were automatically collided using helium gas and subjected to MS/MS analysis. The scan range of product ions was from *m*/*z* 0 to 600. Ion spray voltage was set to 4000 V. Capillary temperature was kept at 350 °C. Capillary voltage was 25 V with tube lens offset 80 V. Nitrogen was used as sheath gas and auxiliary gas, and the rate of flow was set to 35 arb and 5 arb (1 arb = 0.3 mL·min^−1^), respectively.

### 3.4. UPLC-TOF MS Instrumentation

Further confirmation by TOF MS was performed on Agilent 1290 Infinity LC system coupled with Agilent 6538UHD Accurate-Mass Q-TOF mass spectrometer (Agilent, Santa Clara, CA, USA). Data acquisition and processing were carried out using Agilent MassHunter Workstation Software (Version B 06.00). The chromatographic conditions were as follows: An ACQUITY UPLC HSS T3 column (2.1 mm × 100 mm, 1.8 µm; Waters, Milford, MA, USA); sample injection volume, 5 µL; Temperature of column oven, 40 °C; flow rate, 0.4 mL·min^−1^. The mobile phases were 0.1% (*v*/*v*) formic acid (A) and acetonitrile (B). A gradient elution was used as follows: 0–3 min, 15% B; 3–8 min, 15%–20% B; 8–12 min, 20%–30% B; 12–15 min, 30% B. The optimized TOF MS conditions were as follows: capillary voltage, 4.0 kV; drying gas flow, 11 L/min; gas temperature: 350 °C; nebulizer pressure,45 psi; fragmentor voltage, 120 V; skimmer voltage, 60 V. Full-scan data acquisition was performed from *m*/*z* 100 to 1000 in centroid in positive mode.

Urine samples were prepared through normal SPE procedure using a Waters Oasis HLB (3cc/60 mg, Waters) SPE column. 1 mL urine sample was loaded and subjected to wash procedure using 3 mL water. After that, the components in SPE column were eluted using 3 mL methanol. The collected eluent was evaporated to dryness and resolved in 100 µL 80% methanol prior to UPLC-TOF MS analysis.

### 3.5. Preparation of Major Metabolites

Urine samples were thawed under room temperature, vortexed and centrifuged under 3000 rpm for 10 min. 5 mL solution of β-glucuronidase was added to 300 mL supernatant under 37 °C for hydrolysis reaction lasting 4 h. The hydrolytic solution was then vortexed and centrifuged under 3000 rpm for 10 min prior to microporous resin purification.

The purification step was conducted using a glass column (80 cm × 4 cm i.d.) packed with D-101 macroporous resin. The bed volume was approximately 600 mL. 300 mL urine sample was loaded at the flow rate of 10 mL·min^−1^. After the solvent in column has been replaced by urine samples, the resin was allowed for absorption equilibrium for one hour. The column was firstly washed using 1500 mL deionized water at the flow rate of 10 mL·min^−1^ to remove endogenous components of high polarity in urine. After that the column was washed using 300 mL 40% (*v*/*v*) ethanol, and then subjected to desorption elution using 2000 mL 95% (*v*/*v*) ethanol. The collected eluent was pooled, concentrated to dryness and resolved in 50 mL 50% (*v*/*v*) acetonitrile for further PHPLC preparation.

The preparation step was accomplished on an identical instrumental system mentioned in Section 3.3 except for a sample loop with larger volume (3 mL). Chromatographic separation was conducted using an YMC-Pack Pro C18 RS column (150 mm × 10.0 mm i.d., 5 µm, 8 nm, YMC Co., Ltd.) at 45 °C, and the detection wavelength was 281 nm. The mobile phase in isocratic mode consisted of 35% Acetonitrile and 65% water (containing 0.1% Formic acid). Preparation of single injection was completed in 10 min.

### 3.6. Nuclear Magnetic Resonance Analysis

The isolated metabolites M4 and M5 were subjected to ^1^H-NMR and 13C-NMR analysis on a Avance II 600 MHz NMR spectrometer (Bruker, Faellanden, Switzerland) with deuterated methanol (MeOD) as solvent.

## 4. Conclusions

The proposed online SPE-HPLC-MS^n^ method in this study was successfully applied to detect the metabolites in human urine after intravenous administration of Pyragrel, a new synthetic platelet aggregation inhibitor. Five metabolites were detected and tentatively identified with their molecular structures inferred based on the fragmentation patterns. The proposed major metabolic pathways of Pyragrel in human urine were double-bond reduction, unsaturated fatty acid oxidation, followed by glucuronide conjugation. Two main metabolites, M4 and M5 were then prepared using β-glucuronide hydrolysis and macroporous resin purification approach followed by preparative high-performance liquid chromatography (PHPLC) method, with their structures confirmed on the basis of nuclear magnetic resonance (NMR) data. To sum up, this study provided information for the further study of the metabolism and excretion of Pyragrel.

## Figures and Tables

**Table 1:** Tandem mass spectrometry (MS^n^) data of Pyragrel and its metabolites in human urine.

No.	t_R_ (min)	Parent Ions ([M + H]^+^, *m*/*z*)	MS^n^ (*m*/*z*)	Accurate Ion (*m*/*z*) and Deduced Molecular Formula by TOF MS	Reaction Type
M1	4.23	479	MS^2^: 303(100) MS^3^: 285, 259, 136	479.1666; C_22_H_26_N_2_O_10_	glucuronidation
M2	5.53	319	MS^2^: 301(100), 275, 151 MS^3^: 283, 260, 151	319.1293; C_16_H_18_N_2_O_5_	oxidation
M3	11.57	347	MS^2^: 329(100), 311 MS^3^: 311, 269	347.1605; C_18_H_22_N_2_O_5_	oxidation
M4	14.03	303	MS^2^: 285, 259, 151, 136(100) MS^3^(285): 257, 244	303.1350; C_16_H_18_N_2_O_4_	double-bond oxidation
M5	19.59	331	MS^2^: 313(100) MS^3^: 298, 271, 191, 135	331.1659 C_18_H_22_N_2_O_4_	double-bond reduction
Pyragrel	20.10	329	MS^2^: 311(100), 285, 177 MS^3^: 296	329.1501 C_18_H_20_N_2_O_4_	

**Table 2:** Gradient elution and valve switching programs.

SPE Pump (Left Pump)				Analytical Pump (Right Pump)				Valve	
Time (min)	Flow rate (mL/min)	Solvent A ^a^ (%)	Solvent B ^b^ (%)	Time (min)	Flow Rate (mL/min)	Solvent A ^a^ (%)	Solvent B ^b^ (%)	Switch Time (min)	Valve State
0	1	100	0	0	1	85	15	0	1-2
0.5	1	85	15	5	1	85	15	0.5	6-1
1	0.3	10	90	15	1	80	20	1	1-2
25	0.3	10	90	20	1	70	30		
26	1	100	0	25	1	70	30		
30	1	100	0	25.1	1	85	15		
				30	1	85	15		

^a^ water, containing 0.4% acetic acid and 25 mM ammonium acetate; ^b^ acetonitrile.

## Supplementary Materials

Supplementary materials are available online.
