# Supplementary material for: Profiling and Preparation of Metabolites from Pyragrel in Human Urine by Online Solid-Phase Extraction Coupled with High Performance Liquid Chromatography Tandem Mass Spectrometry Followed by a Macroporous Resin-Based Purification Approach
Source: Molecules. 2017 Mar 21;22(3):494. doi: 10.3390/molecules22030494 (PMC6155396; doi:10.3390/molecules22030494)
Supplement: Supplementary file 1 [file molecules-22-00494-s001.pdf]

Profiling and Preparation of Metabolites from Pyragrel in Human Urine by Online Solid-Phase Extraction Coupled with High Performance Liquid Chromatography Tandem Mass Spectrometry Followed by a Macroporous Resin-Based Purification Approach

Xin Zhao [1,†], Jingjing Jiang [2,†], Guang Yang [2], Jie Huang [3], Guoping Yang [3], Guangwei He [4], Zhaoxing Chu [4], Taijun Hang [1,*] and Guorong Fan [2,5,6,*]

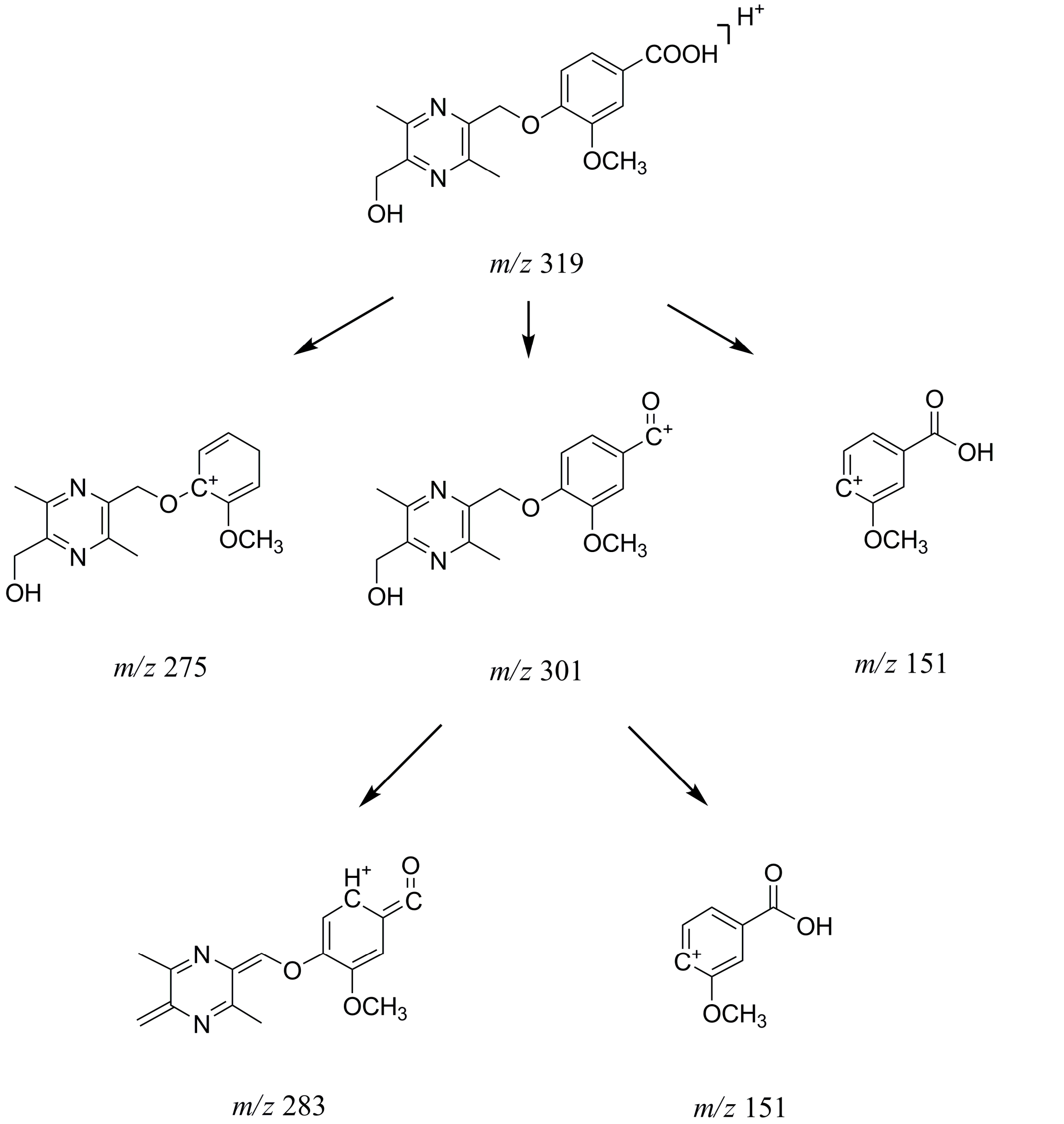

Fig. S1 Inferred fragmentation patterns of metabolite M2.

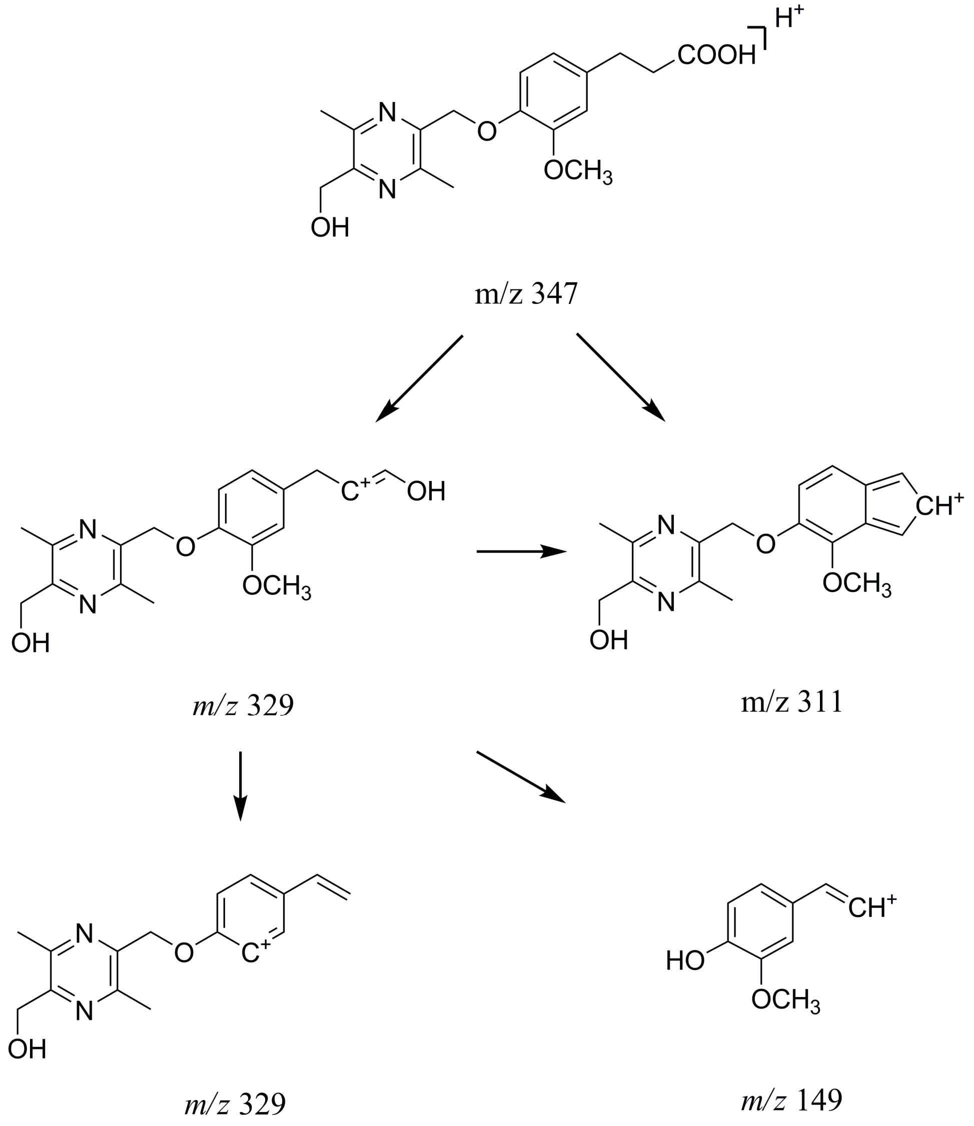

Fig. S2 Inferred fragmentation patterns of metabolite M3.

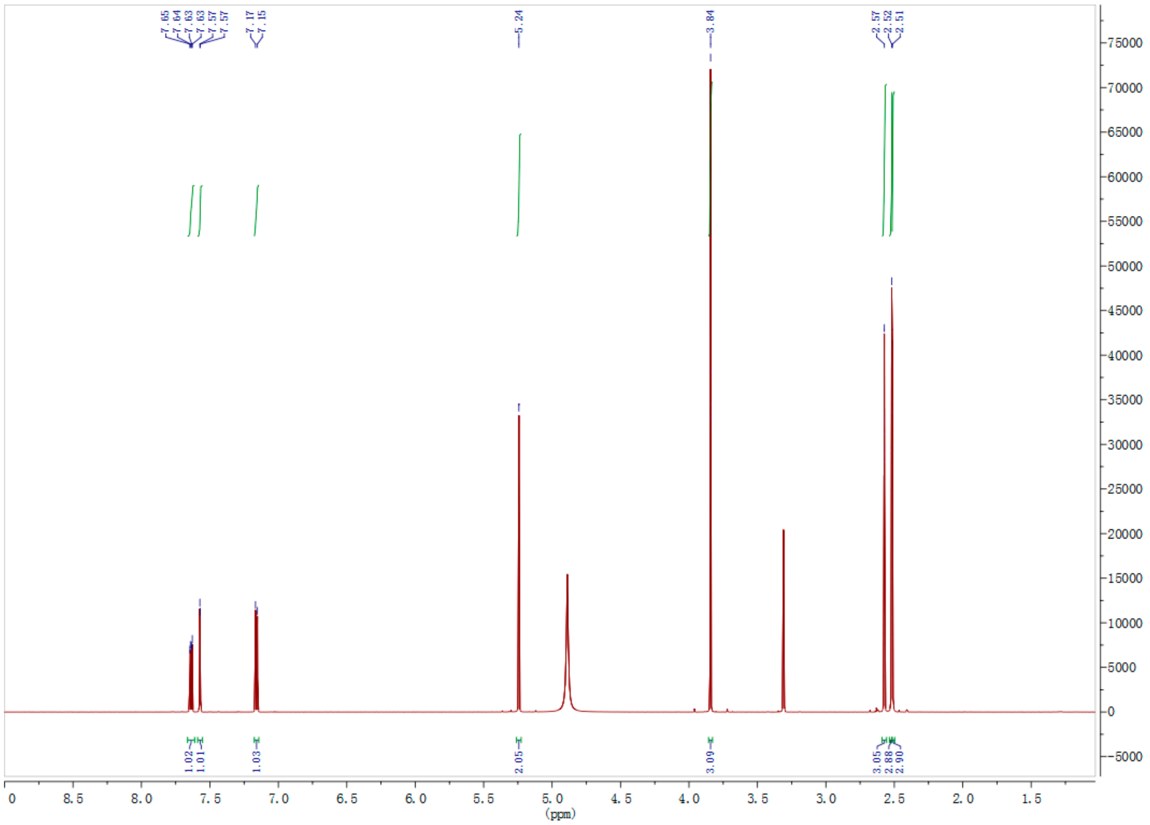

Fig. S3 ^{1}H NMR spectrum of metabolite M4.

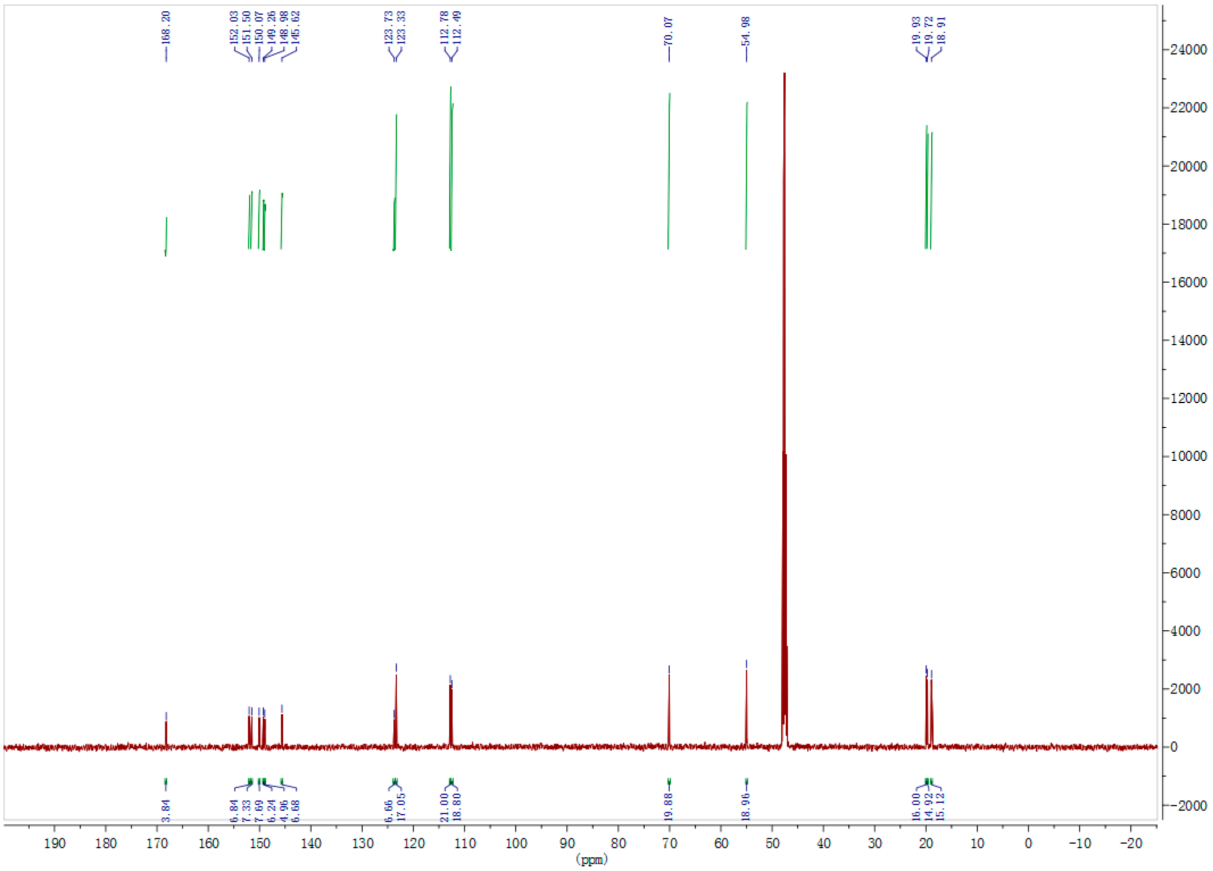

Fig. S4 ^{13}C NMR spectrum of metabolite M4.

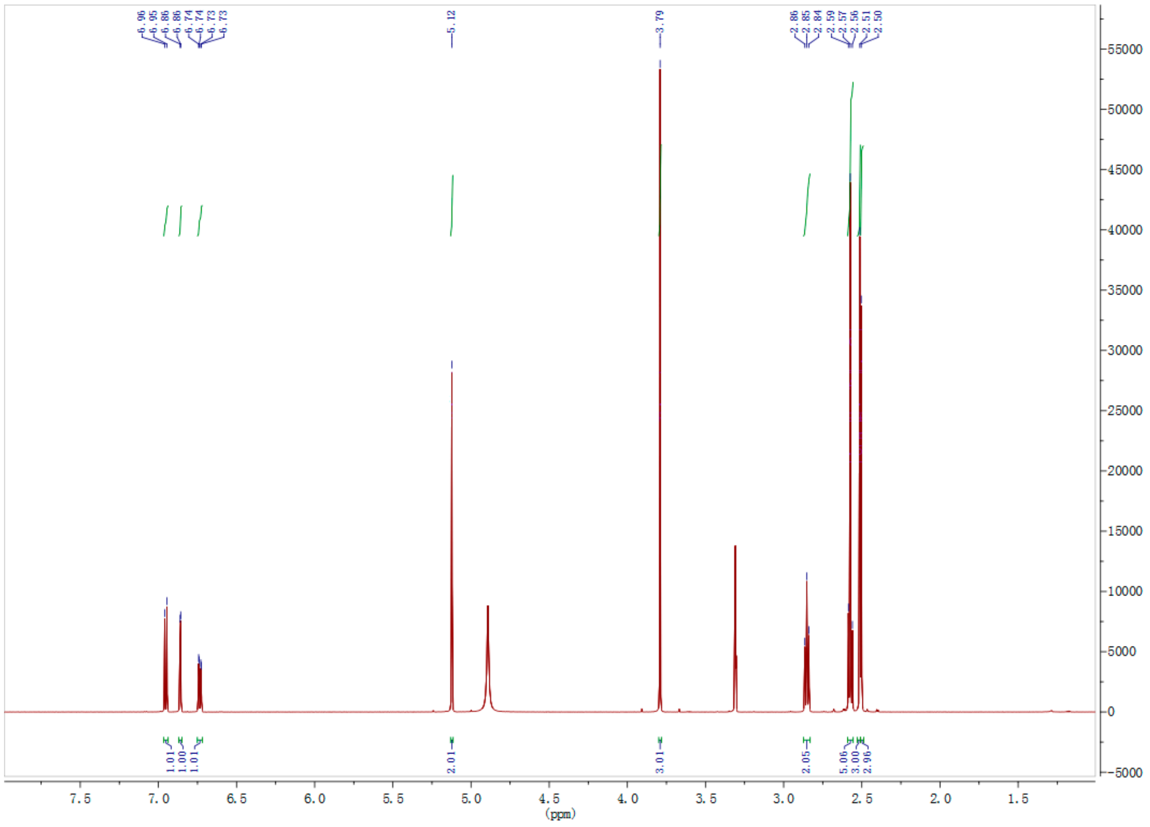

Fig. S5 ^{1}H NMR spectrum of metabolite M5.

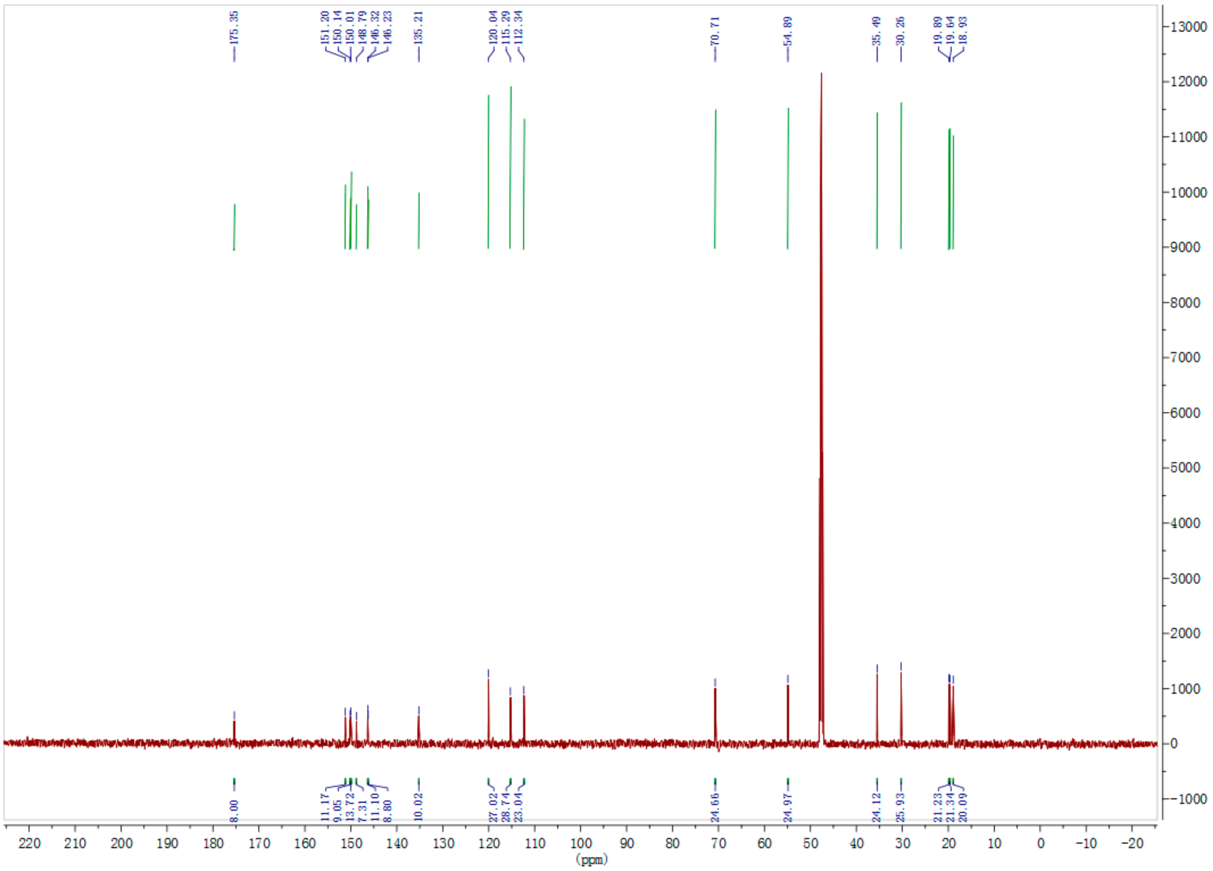

Fig. S6 ^{13}C NMR spectrum of metabolite M5.
